# Initial success of native grasses is contingent on multiple interactions among exotic grass competition, temporal priority, rainfall and site effects

**DOI:** 10.1093/aobpla/plu081

**Published:** 2014-12-05

**Authors:** Truman P. Young, Emily P. Zefferman, Kurt J. Vaughn, Stephen Fick

**Affiliations:** 1 Department of Plant Sciences, University of California, Davis, CA 95616, USA; 2 Audubon California, PO Box 733, Winters, CA 95694, USA

**Keywords:** Assembly, community structure, exotics, grassland/prairie restoration, invasives, priority effects, site effects, weeds.

## Abstract

Ecological communities are increasingly being recognized as the products of contemporary drivers and historical legacies that are both biotic and abiotic. In an attempt to unravel multiple layers of ecological contingency, we manipulated (i) competition with exotic annual grasses, (ii) the timing of this competition (temporal priority in arrival/seeding times) and (iii) watering (simulated rainfall) in a restoration-style planting of native perennial grasses. In addition, we replicated this experiment simultaneously at three sites in north-central California. Native perennial grasses had 73–99 % less cover when planted with exotic annuals than when planted alone, but this reduction was greatly ameliorated by planting the natives 2 weeks prior to the exotics. In a drought year, irrigation significantly reduced benefits of early planting so that these benefits resembled those observed in a non-drought year. There were significant differences across the three sites (site effects and interactions) in (i) overall native cover, (ii) the response of natives to competition, (iii) the strength of the temporal priority effect and (iv) the degree to which supplemental watering reduced priority effects. These results reveal the strong multi-layered contingency that underlies even relatively simple communities.

## Abstract (precis)

Throughout the western United States, native perennial grasses are being supplanted by aggressive non-native annuals. We show that giving native grasses just a two-week germination 'head start' over exotic invasive grasses shifts the competitive edge strongly in their favour. We also show that the strength of this advantage differs strikingly depending on the site where the experiment is carried out, and the weather in the initial weeks of the experiment. These results a) give insight into the reasons for the competitive advantage that annuals usually demonstrate, and b) are an example of the likelihood that ecological experiments often produce results that are limited to a particular time and place, and less general than we might wish to believe.

## Introduction

Ecological interactions are increasingly recognized as being highly contingent on their context, shaped by forces that are both historical and contemporary as well as biotic and abiotic (Chamberlain *et al.* 2014). For example, variation between years and sites (‘year effects’ and ‘site effects’) can have profound influences on the outcomes of field experiments in community ecology (Bakker *et al.* 2003; Vaughn and Young 2010). If we want the results of ecological experiments to be general, and not unique to a particular site or time, we need to better explore and understand these and other contingencies.

Understanding such contingencies is also crucial for successfully restoring ecosystems. One emerging theme is the phenomenon of priority—how differences in arrival times by different species may have profound effects on the long-term trajectories of communities (e.g. Hoelzle *et al.* 2012; Vannette and Fukami 2014). Such priority effects were the centerpiece of initial definitions of assembly theory, and are currently being explored as potential management techniques in ecological restoration, in particular to assist the establishment of less-competitive species in communities (Hobbs and Suding 2008; Porensky *et al.* 2012).

A number of experimental studies on perennial herbaceous plants have shown that a 1- to 3-week priority can significantly affect initial community structure (Deering and Young 2006; Abraham *et al.* 2009; Grman and Suding 2011; Stevens and Fehmi 2011; Dickson *et al.* 2012; Cleland *et al.* 2014). In other words, initial community structure is contingent on the relative arrival times of species. This includes research in our study system (interior California prairie), where we have extended this concept to show that even small initial priority effects of native perennial grasses over exotic annual grasses can multiply over several years to result in substantially greater cover by the natives (Vaughn and Young 2015).

Priority effects may be particularly relevant for testing the mechanisms underlying the competitive advantage of invasive annual plants over native perennials. In many western US ecosystems, these invasives have become community dominants (Stromberg *et al.* 2007). It has been posited that this competitive advantage is driven by the earlier germination and initially higher growth rates of the annuals (Jackson and Roy 1986; Dyer *et al.* 2000; Rice and Dyer 2001; Harmon and Stamp 2002; Verdu and Traveset 2005; Lulow 2006). Several short-term priority experiments suggest that this is the case (Deering and Young 2006; Abraham *et al.* 2009; Grman and Suding 2011; Cleland *et al.* 2014). Most of these studies were carried out at a single site and in a single planting year, and we do not know how the strength and consequences of this priority effect differ though space and time.

The structure of communities may also be dependent on conditions in the year in which they were established (e.g. Chamberlain *et al.* 2014). Ecologists (Bakker *et al.* 2003; MacDougall *et al.* 2008; Seabloom 2011), and restoration practitioners (J. Anderson, pers. comm.) have noted differences in project outcomes and results from experiments initiated in different years, but these have not been subject to controlled experiments where putative drivers of year differences are manipulated.

Community structure may be also contingent on site conditions, and the relative abundances of different species may change over relatively small environmental gradients (Grman *et al.* 2013). It is likely that these differences are due to a combination of site effects, year effects (Knappová *et al.* 2013) or differences in restoration practices (Grman *et al.* 2013), but these different factors have rarely been examined together in controlled, replicated experiments.

Here we report the results of experimental tests of how seeded native perennial grass cover is influenced by (i) competition with exotic annual grasses, (ii) the relative timing of seed arrival (temporal priority effect), (iii) rainfall addition and (iv) geographical location (site effect). We also tested the interactions among priority, rainfall addition and site effects.

## Methods

At three different sites, we manipulated the timing of competition between native perennial grasses and exotic annual grasses (priority effect) with four different planting treatments, and crossed these with two watering treatments to simulate rainfall differences between years.

### Study sites

In November 2011, we established a set of experimental plots as part of a long-term study of priority effects, site effects and rainfall in the context of grassland/prairie restoration. The entire experiment is replicated over three sites in north-central California which have similar relatively fertile clay loam soils, but differ moderately in elevation and climate (temperature and rainfall), and weed challenge (Table 1). All had been used for (different types of) crop agriculture in the past, but had been fallow for several years before the experiment, and were dominated by exotic weeds before site preparation.

**Table 1.** Site characteristics. The reported temperatures are mean (2001–12) daytime highs during the early growing season and the height of the growing season.

	Davis research fields	Hopland Research and Extension Center	McLaughlin Natural Reserve
Latitude	38°32′N	39°00′N	38°52′N
Longitude	121°51′W	123°04′W	122°25′W
Elevation (m)	15	150	650
Mean annual/December rainfall (mm)	470/80	870/200	730/140
2011/12 Total/December rainfall (mm)	284/8.5	232/5.0	539/1.5
Mean max Nov/max March (°C)	17.9/18.8	18.1/18.1	14.8/14.8
Soil	Brentwood silty clay loam	Cole loam, Feliz clay loam	Yorkville variant clay loam
Weed challenge	Mostly annual forbs, including *Malva parviflora*	Annual forbs, including starthistle; annual grasses, including *B. hordeaceus*.	Annual forbs, annual grasses, including *B. hordeaceus* and *Avena barbata*

### Experimental design

Over the previous 6 months (March–September 2011), we had collected seeds of local provenance at each of the three sites (where possible) from four native perennial grasses and four exotic annual grasses (Table 2). For a few of these 24 provenances for which local reproductive populations could not be located, we purchased seeds from local native seed providers. We made some adjustments at the species level to match local sites: for the annual *Avena* species, we collected and sowed *A. fatua* in Davis and the very similar *A. barbata* at McLaughlin and Hopland; for the annual *Vulpia* species, we collected and sowed *V. myuros* at Davis and McLaughlin, and the similar species *V. bromoides* at Hopland.

**Table 2.** The grass species used in this experiment, and their seeding rates (seeds m^−2^).

Native perennial grasses	Exotic annual grasses
*Stipa (Nassella) pulchra* (100)	*Vulpia (Festuca) myuros/V. bromoides* (Hopland) (400)
*Bromus carinatus* (100)	*B. hordeaceus* (400)
*Hordeum brachyantherum* (100)	*Hordeum murinum* (100)
*Elymus glaucus* (100)	*A. barbata/A. fatua* (Davis) (100)

At each site, we established five blocks, each with two replicates of the following four planting treatments: (i) natives sown alone (N), (ii) natives sown together with exotics (NE), (iii) natives sown and exotic sown 2 weeks after the next germinating rain (NtE) and (iv) exotics sown alone 2 weeks after the next germinating rain (tE). Planting treatments were implemented in an additive design (for seeding densities, see Table 2). In a split-plot design, blocks were divided in half, with one half designated for rainfall manipulation (one replicate of each planting treatment was randomly located within each half-block). Each experimental plot was 1.25 m on a side, and each was separated from adjacent plots by 1 m.

Prior to planting, all sites were tilled to control weeds, both before and 1–2 weeks after the first germinating rains in the fall. Within 1 week of the second tilling, we did the first sowing (18–20 November 2011). Each plot was lightly raked, sown and then raked again to increase seed–soil contact. There was a second germinating rain on 24 November. Two weeks later, the plots designated to receive a second sowing (of exotics) where sown. Unusually, there had been little rain in the intervening 2 weeks, and there was no rain in the 5 weeks that followed the second sowing. Therefore, to simulate an early-season rain that was more similar to a normal year, the four treatments designated for rainfall manipulation (‘Watering’ treatment) in each block were watered with the equivalent of 1.25 cm of rain immediately after the second sowing pass (5–7 December).

Over the following weeks, plots were weeded of volunteer forbs. Because grasses are difficult to reliably identify at the seedling stage and because there were volunteer seedlings of sown species at two of the three sites (see Table 1), we only weeded the obvious non-sown grass species. The result was that all plots had some background of non-sown individuals. Nonetheless, there were significantly greater exotic grass densities in the plots deliberately sown with exotics than in those without (73.5 % cover vs. 26.5 % cover across all three sites; *P* < 0.001).

Surveys were carried out after the main winter rain had ceased in the spring, at the time of peak flowering. For the Davis and Hopland sites, this was 26–31 May 2012. The phenology of the grasses was delayed at the higher elevation McLaughlin site, which was surveyed 8 June 2012. The areal cover of each seeded species was visually estimated for each plot. We also recorded the cover of common non-sown exotic grasses.

### Statistical analyses

For each of the following analyses, linear mixed-effects models were specified with the lme() function from the R software (R Core Team 2012) package ‘nlme’ (Pinheiro *et al.* 2013). Block was included in all of the models as a random effect. Where necessary, variance structures were specified using the VarIdent() function to address violations of homogeneity of variance (Zuur *et al.* 2009). ANOVA tables were generated by calling the anova() command from the ‘stats' package (R Core Team 2012). Due to the nested nature of the design we tested the effects of each factor with sequential sums of squares.

#### Suppression of natives by exotics

To determine whether the presence of exotics affected the growth of natives, and whether these effects differed by site, we tested the effects of site, planting treatment and their interaction on native grass cover in two planting treatments: natives seeded alone (N) vs. natives seeded together with exotics (NE). Only unwatered plots were included in this analysis.

#### Priority, site and simulated year effects

To determine the effects of priority, watering and site on native and exotic cover, we compared two planting treatments—natives and exotics seeded together (NE) and exotics seeded after natives (NtE). Planting treatment, watering treatment, site, and all two-and three-way interactions were included in models of native cover and exotic cover (separately). Priority treatment was nested in watering treatment to account for the split-plot design. Due to large and significant site effects and priority treatment interactions with site, we also analysed the same dataset for each site separately.

## Results

### Suppression of natives by exotics

**Figure 1.**
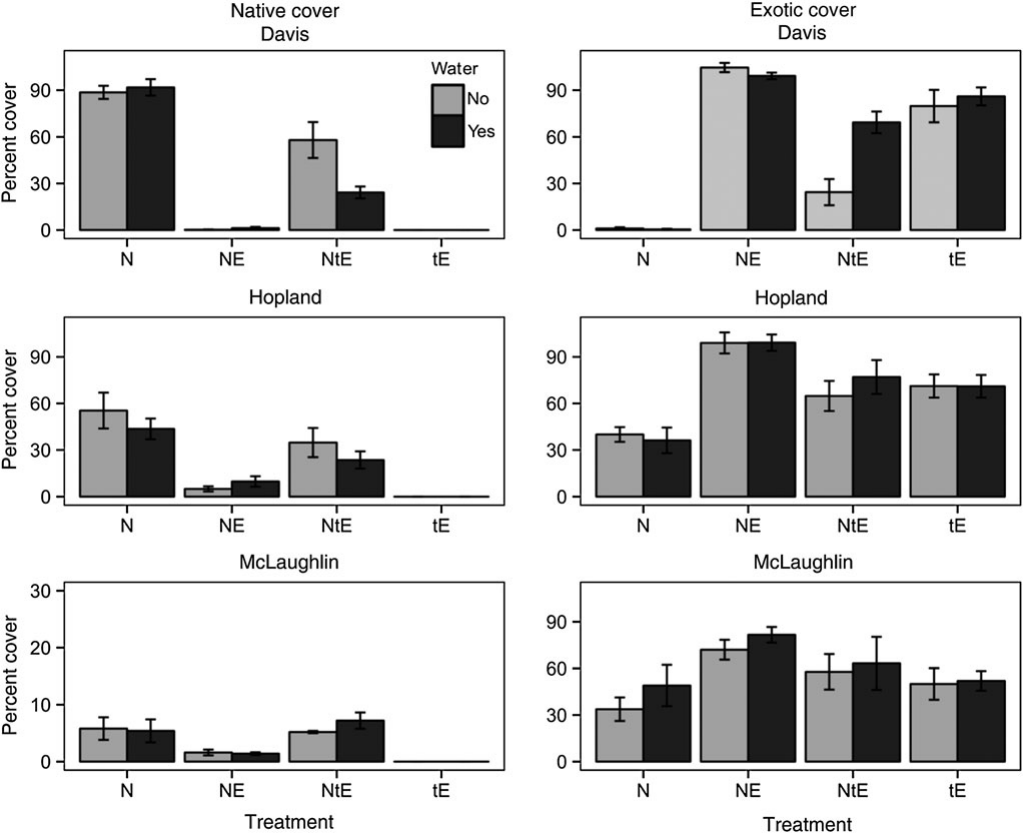
Native perennial grass cover (left) and exotic annual grass cover (right) in different experimental treatments in each of the three experimental sites. N, natives seeded alone; NE, natives and exotics seeded together; NtE, exotics seeded 2 weeks after the natives; tE, exotics seeded alone, 2 weeks after the initial seedings. Bars are one standard error. Note the different scale used for native cover for the McLaughlin plots.

**Figure 2.**
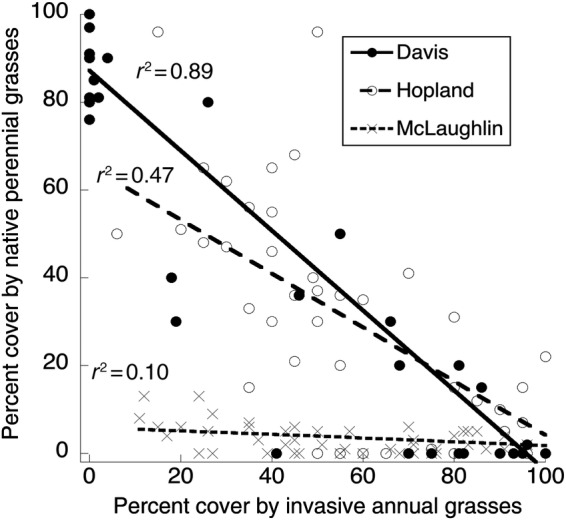
Relationship between per cent cover by exotic annual grasses and per cent cover by native perennial grasses within each site, across all plots. All three correlations are statistically significant, as is the overall correlation.

The abundance of native grasses at the end of the first growing season differed significantly by site (Fig. 1, Table 3, *P* < 0.002). At the coolest site (McLaughlin) the sown natives (and exotics) achieved the lowest total cover. Exotic annual grasses greatly suppressed the native perennials in all the three sites. When the native perennial grasses were sown together with exotic annual grasses, they achieved 73–99 % less cover than when seeded alone (N vs. NE, Fig. 1, Table 3, *P* < 0.0001). These differences among sites were themselves significant, with the site that had lower cover overall showing less suppression of natives by exotics (N vs. NE, site × competition interaction, Table 3, *P* < 0.0001). Across all treatments and sites, cover by seeded exotic annual grasses was strongly negatively correlated with cover by seeded native perennial grasses at the plot level (*r*^2^ = 0.52, *P* < 0.001, Fig. 2). This correlation was also significant within each of the three sites.

**Table 3.** ANOVA model testing effects of site and competition on native cover for N vs. NE planting treatments, non-watered only. DF, degrees of freedom.

Factor	Num DF	Denom DF	*F*-value	*P*-value
Site	2	12	10.45	0.002
Competition	1	12	129.68	<0.0001
Site × competition	1	12	168.04	<0.0001

### Priority, site and simulated year effects

The analysis of priority effects (natives and exotics seeded together (NE) vs. exotics seeded after the natives (NtE)), and how these priority effects differed between watering treatments and across sites revealed a variety of significant main effects and interactions. First, giving the native perennial grasses a short-term advantage over exotic annual grasses (seeded 2 weeks after the first germinating rains following native seeding) greatly increased their success (Fig. 1, Table 4, priority, *P* < 0.0001). However, this priority effect varied significantly across sites, ranging from 60 to 90 % increases in cover (Table 4, site × priority interaction, *P* < 0.0001). The significant interaction between site and priority was because sites with high cover in general (both natives and exotics) had greater reductions in native cover with competition.

**Table 4.** Results of ANOVA model of site, watering treatment (year effects) and priority (planting treatments = NE vs. NtE) on native cover.

Factor	Num DF	Denom DF	*F*-value	*P*-value
Site	2	12	75.52	0.0024
Water	1	12	2.26	0.16
Priority	1	24	29.98	<0.0001
Site × water	2	12	1.27	0.32
Site × priority	2	24	19.36	<0.0001
Water × priority	1	24	0.07	0.79
Site × water × priority	2	24	5.26	0.013

However, the plants at each site responded differently to the interaction between watering and priority, resulting in a significant three-way interaction (Fig. 1, site × watering × priority interaction, *P* = 0.013, Table 4). At Hopland the muting of priority effects by watering was less than that at Davis, and in McLaughlin, watering even tended to accentuate the effects of priority (Fig. 1; Table 5B and C).

**Table 5.** (A–C) ANOVA results of native cover analysed separately by site (priority = NE vs. NtE). ANOVA with weighted variance (Levene test on data for each site showed significant effects of priority and water).

Factor	Num DF	Denom DF	*F*-value	*P*-value
(A) Davis—native cover				
* *Water	1	4	14.41	0.02
* *Priority	1	8	67.70	<0.0001
* *Water × priority	1	8	8.11	0.02
(B) Hopland—native cover				
* *Water	1	4	1.63	0.27
* *Priority	1	8	18.14	0.003
* *Water × priority	1	8	2.44	0.16
(C) McLaughlin—native cover				
* *Water	1	4	0.02	0.90
* *Priority	1	8	47.89	0.0001
* *Water × priority	1	8	2.62	0.14

The results of the exotic annual grasses were basically mirror images of the results for the native perennial grasses (Fig. 1), with the exotics usually filling in the space not occupied by the seeded natives in each plot. Across all plots, exotic cover was strongly negatively correlated with native cover (Fig. 2). This relationship was statistically significant within each site, but particularly strong at the Davis site (*r* = −0.95, *P* < 0.001), where total cover of all plants was highest. For exotic cover, this correlation was associated with significant site, priority and site × priority effects (Table 6) and, for Davis only, a significant watering × priority interaction (Table 7A).

**Table 6.** Full model: effect of site, watering and priority on exotic cover. ANOVA with a weighted variance structure, where variance is different for each priority (normality was good for exotics, and Levene test only showed significant effects of planting priority).

Factor	Num DF	Denom DF	*F*-value	*P*-value
Site	2	12	11.17	0.002
Water	1	12	1.57	0.23
Priority	1	23	51.75	<0.0001
Site × water	2	12	0.27	0.77
Site × priority	2	23	6.05	0.008
Water × priority	1	23	4.69	0.04
Site × water × priority	2	23	2.95	0.07

**Table 7.** (A–C) Cover by exotics analysed separately by site. ANOVA with weighted variance (Levene test on data from each site showed effects of Priority significant or nearly so).

Factor	Num DF	Denom DF	*F*-value	*P*-value
(A) Davis—exotic cover				
* *Water	1	4	0.01	0.93
* *Priority	1	8	90.83	<0.0001
* *Water ×priority	1	8	19.07	0.002
(B) Hopland—exotic cover				
* *Water	1	4	0.20	0.68
Priority	1	8	11.10	0.01
Water × priority	1	8	0.50	0.50
(C) McLaughlin—exotic cover				
Water	1	4	1.38	0.30
Priority	1	8	3.99	0.09
Water × priority	1	8	0.03	0.86

## Discussion

It is not surprising that the success of sown native grasses was greatly reduced when sown together with exotic annual grasses (Fig. 1, N vs. NE), and that in general, cover by exotic annual grasses and native perennial grasses were strongly negatively correlated (Fig. 2). In grassland restoration projects in the Central Valley of California, the presence of exotic annuals represents perhaps the greatest challenge to successfully establishing native perennial grasses, and aggressive pre-sowing control of exotics is now considered a *sine qua non* for restoration. Conversely, one of the most effective means of preventing the dominance of exotic annuals is the establishment of cover by native perennial grasses (see also Tognetti and Chaneton 2012). Together, these processes result in strong negative correlations between exotic and native grasses.

The magnitude of the competitive suppression of natives by exotics, however, varied across the three sites. Site effects are a complex array of interacting differences, including different means and patterns of rainfall and temperatures, different intensities and identities of weed challenge, and different herbivore pressures. We can only suggest which are the important drivers, but note that in the coolest site (McLaughlin), where native grasses achieved little cover in the first year even when planted alone (Fig. 1), they were significantly less affected by the sown exotic annual grasses (Fig. 2), which also had reduced cover (Table 4, site × priority interaction).

Although in practice weed control often seeks to greatly reduce the challenge of exotic annuals for at least the first year of native planting, our results show that even a much briefer respite can have a profound effect. When exotic annual grasses were seeded just 2 weeks after germinating rains for the natives, their ability to suppress these natives was greatly reduced (Fig. 1, NE vs. NtE and priority effect, Table 4). This provides experimental support for the suggestion that one of the ways the exotic annual species outcompete natives in California grasslands is their demonstrated earlier germination and faster growth (see also Deering and Young 2006; Wainwright *et al.* 2011; Vaughn and Young 2015). The fact that the tE treatment had nearly as much exotic cover as the NE treatment (Fig. 1) strongly suggests that the late sowing did not itself greatly reduce eventual exotic cover, but that this occurred only in the presence of natives, i.e. as a priority effect. There are also reasons to believe that these differences in community structure arising from initial differences in our experimental treatments have long-term consequences (Hoelzle *et al.* 2012; Vaughn and Young 2015).

Vannette and Fukami (2014) made several predictions about the strength of priority effects that apply in this system (see Vaughn and Young 2015). In particular, they suggested that priority effects would be greater under higher resource availability (see also Kardol *et al.* 2013). In our system, however, watering reduced the strength of priority effects. This was not because of increased resource availability per se, but rather because the watering treatment effectively reduced the duration of the priority treatment. Greater temporal priority (more days of planting advantage) usually results in stronger priority effects (Kardol *et al.* 2013; Orloff *et al.* 2013; von Gillhaussen *et al.* 2014).

**Figure 3.**
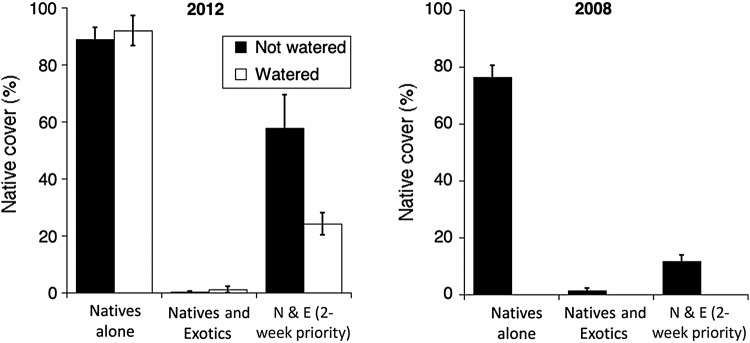
Per cent cover by native perennial grasses across treatments at the Davis site from 2011 to 2012 (left) compared with a similar experiment in 2008–09 (right). Treatments as in Fig. 1. Note that the priority effect in 2008–09 was much more similar to the watered plots in 2011/12 than the unwatered plots. Bars are one standard error. The 2008–09 data were adapted from Vaughn and Young (2015).

This experiment was initiated in a year (2011/12) when there was a 4-week drought following a few weeks of germinating rains in November (Table 1). Our watering treatment suggests that one of the reasons that the priority effect was so strong in our experiment was this early wet season drought that allowed sown natives to grow for almost a full month before exotics germinated. When this drought was partially alleviated by watering, the strength of the priority effect (the difference between native sown at the same time as exotics vs. earlier; Fig. 1) was significantly reduced (Table 5A, priority × watering interaction). We would predict that in a year with more consistent fall rain, these priority effects would be milder. Indeed, in a very similar experiment carried out in 2008, this was the case (Vaughn and Young 2015; Fig. 3).

As with site effects, year effects are likely to be a complex array of interacting differences, including differences in rainfall, temperatures, weed challenges and herbivores. Our watering (equivalent to 1.25 cm of rain) partly offsets the difference between the long-term mean and the rainfall lost during the fall drought of 2011/12, and the result was a priority effect much more similar to 2008 than unwatered 2011/12 (Fig. 3). These results strongly suggest that much of the differences in results between these 2 years can reasonably be attributed to differences in fall rainfall (as opposed to any number of other uncontrolled sources of year effects, such as differences in temperatures, rainfall at other times, pest loads and weed challenge) (see also Reichmann *et al.* 2013).

The three-way interaction (site × water × priority, Table 3) arose because the effect of watering on priority effects differed significantly across sites. Early-season watering during a dry period strongly reduced the benefits of short-term priority for native perennial grasses over exotic annual grasses at Davis, moderately at Hopland and not at all at McLaughlin. We suggest again that the McLaughlin result was related to the overall lower growth at this cooler site, which may have reduced both competition and priority effects, and if so also reduced the effect of watering on this priority effect.

These three sites were chosen to represent characteristic settings for grassland restoration in our area, namely former agricultural plots on alluvial clay loams (Table 1). All three are in interior northern California plant communities within 125 km of each other on similar soils, and the three climates are within the range of projections for medium-term climate change. Recommended native seed mixes for all three are the same except for provenances (H. Farms, pers. comm.). Yet the differences across sites were enough to produce widely divergent covers of restored native grasses and widely divergent responses to our experimental manipulations.

## Conclusions

The fact that the complex interplay between all main effects and their interactions is at least partly explicable (if not predictable) only partly mitigates for their more troubling implications. Our results suggest that not only is the success of restored native perennial grasses significantly affected by site effects and year effects, but that the basic conclusions from experimental manipulations can differ dramatically across relatively similar sites and conservatively simulated year differences.

This also raises the uncomfortable possibility that results of many ecological field experiments initiated in a single site and/or a single year run the risk of being idiosyncratic rather than general (see also Vaughn and Young 2010). This may come as little surprise to restoration practitioners, who have long noticed that restoration outcomes can differ between years and between sites thought to be relatively similar. However, it appears that ecologists still only rarely repeat experiments across multiple years (Vaughn and Young 2010). The results presented here are part of a larger multi-year study that is designed to more fully explore these effects and their implications.

## Sources of Funding

This study was supported by grants from the Elvinia Slosson Endowment and NSF DEB 10-50543.

## Contributions by the Authors

All authors have made a substantial contribution to the manuscript and/or the research presented. T.P.Y. conceived the project and did data collection. T.P.Y., S.F. and K.J.V. implemented the experiment. S.F. and E.P.Z. oversaw seed collection and preparation, and weed control. E.P.Z. and T.P.Y. did statistical analyses. T.P.Y. wrote the first and final drafts. All authors contributed to editing of the manuscript.

## Conflicts of Interest Statement

None declared.
